# Antifouling and Flux Enhancement of Reverse Osmosis Membrane by Grafting Poly (3-Sulfopropyl Methacrylate) Brushes

**DOI:** 10.3390/membranes11030213

**Published:** 2021-03-18

**Authors:** Reema Mushtaq, Muhammad Asad Abbas, Shehla Mushtaq, Nasir M. Ahmad, Niaz Ali Khan, Asad U. Khan, Wu Hong, Rehan Sadiq, Zhongyi Jiang

**Affiliations:** 1 Polymer Research Lab, School of Chemical and Material Engineering, NUST, H-12, Islamabad 44000, Pakistan; rmushtaq_mse11@scme.nust.edu.pk (R.M.); masad_nse02@scme.nust.edu.pk (M.A.A.); shehla.mushtaq@sns.nust.edu.pk (S.M.); 2 Key Laboratory for Green Chemical Technology of Ministry of Education, School of Chemical Engineering and Technology, Tianjin University, Tianjin 300072, China; wuhong@tju.edu.cn (W.H.); zhyjiang@tju.edu.cn (Z.J.); 3 Department of Chemical Engineering, COMSATS University Islamabad, Lahore Campus, Lahore 54000, Pakistan; asadkhan@cuilahore.edu.pk; 4 School of Engineering, University of British Columbia (Okanagan), 3333 University Way, Kelowna, BC V1V 1V7, Canada; rehan.sadiq@ubc.ca

**Keywords:** antifouling, PSMPK brushes, grafting, ATRP, *E. Coli*, *S. Aureus*, TFC-PA RO membrane

## Abstract

A commercial thin film composite (TFC) polyamide (PA) reverse osmosis membrane was grafted with 3-sulfopropyl methacrylate potassium (SPMK) to produce PA-g-SPMK by atom transfer radical polymerization (ATRP). The grafting of PA was done at varied concentrations of SPMK, and its effect on the surface composition and morphology was studied by Fourier-Transform Infrared Spectroscopy (FTIR), Scanning Electron Microscopy (SEM), optical profilometry, and contact angle analysis. The grafting of hydrophilic ionically charged PSPMK polymer brushes having acrylate and sulfonate groups resulted in enhanced hydrophilicity rendering a reduction of contact angle from 58° of pristine membrane sample labeled as MH0 to 10° for a modified membrane sample labeled as MH3. Due to the increased hydrophilicity, the flux rate rises from 57.1 L m^−2^ h^−1^ to 71.2 L m^−2^ h^−1^, and 99% resistance against microbial adhesion (*Escherichia coli* and *Staphylococcus aureus*) was obtained for MH3 after modification

## 1. Introduction

The population of the world is exponentially increasing day by day. Demand for the fresh water increases with the rise in population rate and industrialization [1]. Among major health-related issues, poor quality of the drinking water infected with various contaminants is one of the crucial challenges nowadays [2]. Well-known contaminants in the drinking water are bacteria, viruses, pesticides, toxic metals, fertilizers, industrial effluents, and organic matter, leading to epidemic and major water-borne diseases [3]. Different approaches have been employed for water disinfection from a variety of contaminants in the past few decades such as precipitation and coagulation, distillation, adsorption, ion exchange, catalytic processes, bioremediation, magnetic separation, and membrane water treatment technologies [4,5,6]. Membrane technology has been commonly used for various water treatment applications because of its compactness, flexibility, high efficiency, low operational cost, and simplification of process, rejecting several contaminants that range from microns to angstroms [7].

A selective thin PA film (≈100 nm) fabricated on top of a macro-porous support through interfacial polymerization (IP) plays a promising role in the membrane desalination process [8]. It is a physical barrier that permits certain entities such as solvents to pass while blocking the impurities. The thin film composite (TFC) reverse osmosis (RO) membrane has good water permeability, lower salt discharge, compaction tolerance, large operating temperature, and pH ranges relative to other RO membranes [9]. The fouling of TFC membranes remains a crucial issue despite the wide array of uses for marine and brackish water wastewater treatment and reuse of wastewater [4]. Foulants in feed water develop a film on the membrane surface, contributing to a reduction in water flow and permeate consistency [10]. Therefore, there is a need for the control of fouling on the membrane surface, and significant efforts has been made to develop efficient methods for prevention of formation of foulant film on the membrane [11]. Improved surface hydrophilicity is generally accepted to decrease the fouling risk due to the creation of a dense hydration layer, which is a strong barrier to foulant adsorption. [12]. Therefore, the adaptation of the membrane surface quality by surface tailoring should be an efficient approach to raise the antifouling characteristics of the membrane [13]. However, several studies have documented that the grafting of hydrophilic materials results in increased surface hydrophilicity by an antifouling alteration of TFC membranes [13]. One of the most commonly used antifouling agents is poly ethylene glycol (PEG), which is very productive in enhancing the surface hydrophilicity by attracting H_2_O molecules through hydrogen bonding, resulting in decreased organic foulant adhesion [11]. Despite its extensive use, PEG drops its antifouling features as a result of oxidation in complex media [14]. The grafting of hydrophilic nanomaterials, such as silica nanoparticles, graphene oxide, and carbon nanotubes on TFC-PA membranes, has exhibited improved surface hydrophilicity, showing varying degrees of antifouling efficiency [15]. Polymer brushes have been granted significant attention lately because of their strong hydrophilicity, long-term resilience, and environmental safety as potential antifouling materials [12]. Wagner et al. studied that the grafting of poly ethylene glycol diglycidyl ether (PEGDE) on the top of the TFC-PA membrane reduces the fouling results from charged surfactants such as sodium dodecyl sulfate, etc. with the limitation of lower water flux [1]. Cheng et al. reported that the grafting of *N*-isopropyl acrylamide by redox-initiated polymerization following by acrylic acid results in improved hydrophilicity by lowering the contact angle from 52° to 38° with 35 L m^−2^ h^−1^ (LMH) water flux and 97% salt rejection [16]. Patel et al. grafted 3-sulfopropyl methacrylate potassium (SPMK) on poly vinyl chloride (PVC) membrane enhancing water flux from 1.22 L m^−2^ h^−1^ to 3.37 L m^−2^ h^−1^ [17]. Many modification approaches have gained importance recently to fabricate hydrophilic polymer membranes because of their ease of processing and moderate conditions [18]. An efficient method is to synthesize polymer brushes, utilizing a “grafting from” technique via SI-atom transfer radical polymerization (ATRP). [13]. ATRP is performed through a redox active transition metal complex, which reacts with the ATRP initiator in a lower oxidation state and as a result, a free radical is generated, stimulating chain propagation. Deactivation may also take place by reacting with higher oxidation state catalyst [19]. ATRP provides many advantages, such as regulated thickness, mild reaction, and the use of a wide variety of monomers, easy experimental configuration, and flexibility of use in aqueous as well as organic media [19,20,21,22,23]. Patel et al. reported the modification of a pressure-retarded osmosis membrane by grafting copolymer of SPMK with PVC by using ATRP. It showed a water flux of 3.37 L m^−2^ h^−1^ at 14.7 bars, which was greater than the virgin PVC membrane with 0.95 L m^−2^ h^−1^ due to the grafting of a hydrophilic ionic polymer [24]. This shows that water flux improves to a little extent but there are concerns about foulant adhesion in case of fouling.

The above-mentioned studies indicate that the antifouling polymer brushes would be promising antifouling material to be used for tailoring the surface of water treatment membranes. One of the main research challenges that remain is the optimization of antifouling properties of TFC-RO membranes along with high water flux [1,25]. Therefore, the current work is significant and describes the alteration of a TFC-polyamide (PA) RO membrane by incorporating varying concentrations of SPMK to develop high permeability fouling resistant membranes through controlled graft polymerization via ATRP. Different steps involved in the growth of antifouling PSPMK brushes are the functionalization of TFC-PA RO membrane by (3-aminopropyl) trimethoxysilane (APTMS) accompanying the attachment of bromo initiator and polymerization of 3-sulfopropyl methacrylate potassium (SPMK) at radical sites in SI-ATRP. Characterization of modified membrane was carried out at each step by contact angle analysis, FTIR, SEM, and optical profilometry to determine various surface properties such as the wettability, composition, morphology, and roughness. Salt and water permeability tests both for pure and modified membranes were carried out. Antifouling properties of MH membranes after growing PSMK polymer brushes were also investigated.

## 2. Experimental Section

### 2.1. Materials

3-Sulfopropyl methacrylate potassium salt (SPMK, 98%) was used as a monomer, (3-Aminopropyl) trimethoxysilane (97%), Ethyl α-bromoisobutyrate(98%), 2,2ʹbipyridine (99%), Copper-I bromide (CuBr,98%), Copper-II bromide, Dimethylformamide (DMF), and Methanol were purchased from Sigma Aldrich, Darmstadt, Germany. Dow Filmtec BW-30 TFC-PA RO membranes were used as reference membranes for surface modification. All other reagents employed in this experiment were of analytical reagent grade such as nutrient agar (Merck), Triethylamine (TEA), Dichloromethane (DCM), Phosphate buffer saline (PBS), Sodium hydroxide, Paraformaldehyde, and Glacial acetic acid and used as received without further purification. The test strains *Escherichia coli* and *Staphylococcus aureus* were taken from clinical isolates for antibacterial testing.

### 2.2. Methods

#### Modification of Membrane

Prior to grafting, TFC-PA RO membranes were cut into dimensions of (10 × 6) cm^2^ and immersed in deionized (DI) water for about 12 h. After every 1 h, the water was replaced and then completely rinsed with DI water in order to remove all the preservatives on each sample. The procedure is the same as that reported by Kobayashi et al. [26]. Thick sulfonate brushes were grown in three different steps on the surface of the TFC-PA RO membrane. In the first step, the membrane sample was functionalized with a monolayer of 3-aminopropyl trimethoxysilane (APTMS) by soaking it in 0.34 M aqueous APTMS solution for 2 h and then drying it under vacuum for 4 h. Next, an initiator-coated substrate was synthesized by injecting 12.9 µL (0.1 mmol) of ethyl α-bromoisobutyrate and 0.41 mL (3 mmol) of triethylamine in a 60 mL dry DCM under N_2_ atmosphere in a round-bottom flask at 25 °C with a constant stirring for 1 h; then, it was removed, washed with absolute alcohol, and dried under vacuum at 60 °C for 1 h. The third step involves the growth of polymer brushes by dissolving 4.06 mmol SPMK in DMF and water with a ratio of (70:30) in a flask. Degas it by purging it with nitrogen for 20 min. This solution was termed as MH1. Further details of feed ratios for the synthesis of MH2 and MH3 solutions are given in Table 1.

A Copper bipyridine stock solution was prepared by adding 39.6 mg (0.4 mmol) CuBr(I), 53.8 mg CuBr(II), and 312 mg (2 mmol) bipyridine under N_2_. First, 0.5 mL of freshly prepared Cu-Bpy stock solution was injected in a flask containing a homogeneous monomer solution and initiator-immobilized membrane sample. Then, we heated it on a preheated oil bath and stirred it at 300 rpm for 3 h under inert atmosphere for polymerization. Then, we washed the brushes extensively with distilled water, diluted it with HCl to remove K^+^ ions, and dried it under vacuum at 40 °C for two h.

### 2.3. Characterization of Membranes

An Optika B 600 (OPTIKA S.r.l. Ponteranica (BG), Italy) optical microscope was used as an analysis tool to predict the progress of a chemical reaction. The membrane samples are cut into 1 cm^2^ and analyzed by using an optical microscope. BRUKER’s Model ALPHA FTIR (Bruker Optik GmbH Karlsruhe, Germany) spectrometer was employed to analyze the functional groups of membrane samples. The morphological characterization of membrane samples was accomplished by using Scanning Electron Microscopy SEM JOEL JSM-6490A(JEOL, Tokyo, Japan) A Non-contact Optical profilometer NANOVEA PS 50 (Nanovea, CA, USA) was used to quantitatively analyze the surface roughness of membranes. Membrane samples are cut into 1 cm^2^ pieces and then placed on the platform. Data are generated on the attached computer. A sessile drop method was performed with the help of a drop shape analyzer DSA-25 (KRUSS BmbHCo, Germany) to determine the contact angle measurement of a 5 µL deionized H_2_O droplet on a dried membrane sample. The water retention capacity of a modified membrane was carried out by dipping the sample cuttings into H_2_O for 24 h. After drying the membrane cuttings in the vacuum oven for 12 h, the dry weight was calculated, and the water content (%) was calculated by using Equation (1).

(1) $$\%\;Water\;Content=\;\frac{Wet\;Weight-Dry\;Weight}{Wet\;Weight\;}\times 100$$

Permeation of the membrane was performed by measuring the permeate volume in units of liter per square meter per hour (L m^−2^ h^−1^) by using filtrate assembly at a membrane area of 0.025 m^2^ at 1.0 MPa and 25 °C.

#### 2.3.1. Evaluation of Grafting Yield

The grafting degree was calculated gravimetrically by using the weight of a non-grafted membrane sample and the grafted one, as stated in [26]. The membrane samples were completely rinsed with DI water and then dried in the presence of a vacuum at 40 °C until it achieved a constant weight. The grafting yield was determined by Equation (2).

(2) $$Grafting\;Yield\;\left(\%\right)=\frac{W1-W0}{W0}\;\times 100$$

where “*w*_1_” and “*w*_0_” are the constant dry weights of the grafted and ungrafted membrane samples, respectively.

#### 2.3.2. Antibacterial Testing of Membrane Samples

*Staphylococcus aureus* (ATCC 6538) and *Escherichia coli* (ATCC 8739) were employed to test the bacterial efficacy of the modified membrane through the disc diffusion method. Muller Hinton Agar was poured into the Petri plates and allowed to solidify. Membrane samples modified with three different monomer concentrations 4.06 mmol, 8.12 mmol, and 12.18 mmol were cut into 6 mm circular discs and placed on a Petri plate. Cefepime (FEP) was taken as a positive control.

#### 2.3.3. Biofilm Formation Assay

Biofilm formation study was performed against *E. coli* (ATCC 8739) and *Staph.aureus* (ATCC 6538). First, 30 g/L of tryptone soy broth (TSB media) was used as a nutrient solution for culturing bacteria. Prior to use, TSB media was placed in autoclave at 121 °C for 20 min; then, 1 mL of bacterial suspension was added into 30 mL of TSB media following incubation at 37 °C. In case of biofilm formation assay, modified membranes were dipped into freshly inoculated bacterial suspension accompanied by incubation at 37 °C. After 72 h incubation, membrane samples were extracted and first cleaned with 0.1 M PBS solution for 15 min; then, they were rinsed with 4% paraformaldehyde for 30 min to fix the attached bacteria. After fixing, the membranes were washed with 70%, 90%, and 100% ethanol successively several times in order to wash out all the paraformaldehyde. The membranes were dried in air and then observed with SEM JOEL JSM 6490A (JEOL, Tokyo, Japan) for their surface images.

## 3. Results and Discussion

**Figure 1:**
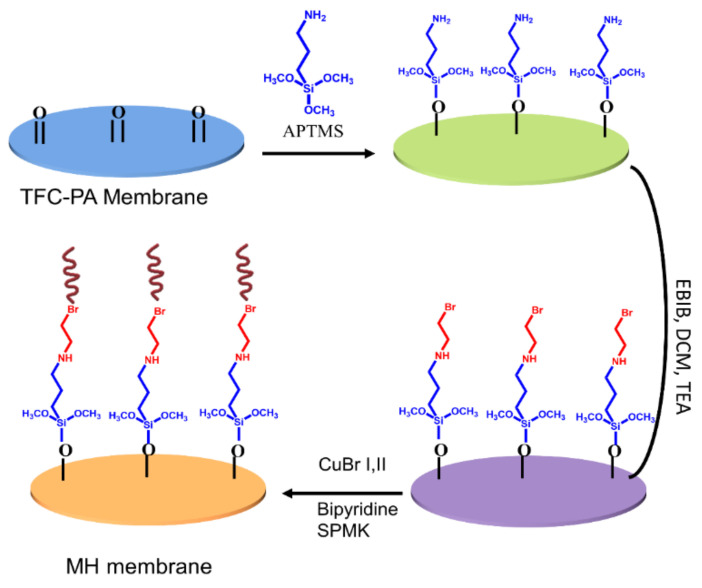
Synthesis of polyamide (PA) g PSPMK brushes via ATRP.

The synthesis scheme for grafting polyamide with 3-sulfopropyl methacrylate potassium through ATRP is displayed in Figure 1. A bottom–up technique i.e., “Grafting from” approach was used in which the PA surface was modified by anchoring a bromo initiator, and then, the polymerization of negatively charged acrylic monomers starts at 40 °C for 3 h in the presence of a catalyst/ligand complex.

It was observed that the grafting yield increases with the increased monomer concentration for the modified membrane samples and found to be 0.75 for MH1, 1.25 for MH2, and 1.90 for MH3. The higher availability of monomer molecules brings about suitable conditions, which assists chain propagation for the grafting of a TFC PA reverse osmosis membrane [27].

### 3.1. Optical Microscopy

**Figure 2:**
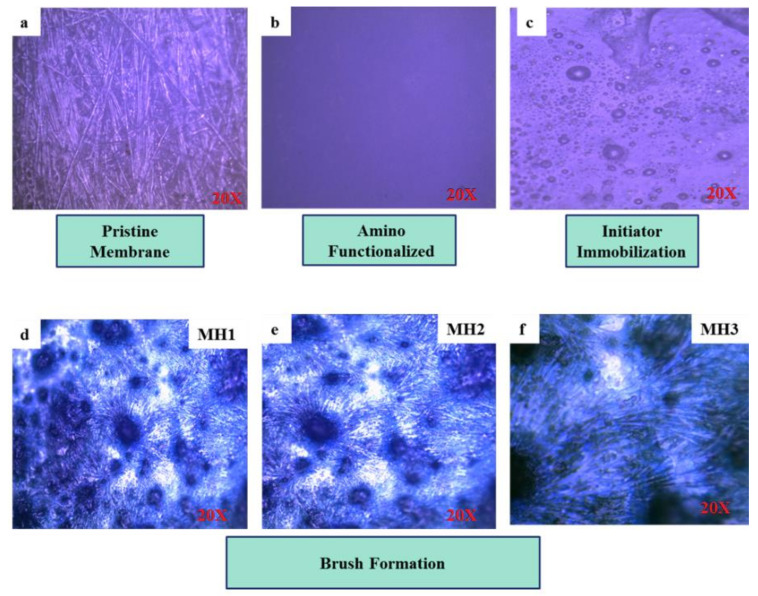
Optical Microscopy of (**a**). Pristine membrane, (**b**) Thin film composite (TFC) PA-NH_2_, (**c**) TFC PA-Br, (**d**–**f**) Modified membranes TFC PA-PSPMK Brushes (MH1, MH2, MH3).

In order to predict the progress of a chemical reaction membrane, samples are analyzed under an Optika B 600 (OPTIKA S.r.l. Ponteranica (BG), Italy) optical microscope. The surface morphology of a pristine reverse osmosis membrane is expressed in labeled Figure 2a,b, which indicates the morphology of the membrane after treating it with APTMS. Figure 2c is obtained after the attachment of an initiator over APTMS functionalized membrane. Modified membranes with grafted polymer brushes at different monomer concentrations are shown in Figure 2d–f. Thus, it has been concluded that polymer brushes have been successfully grown on the TFC-PA membrane by ATRP, as reported in previous studies such as those of Abbas et al. and Wang et al. [28,29].

### 3.2. Functional Group Evaluation by FTIR

**Figure 3:**
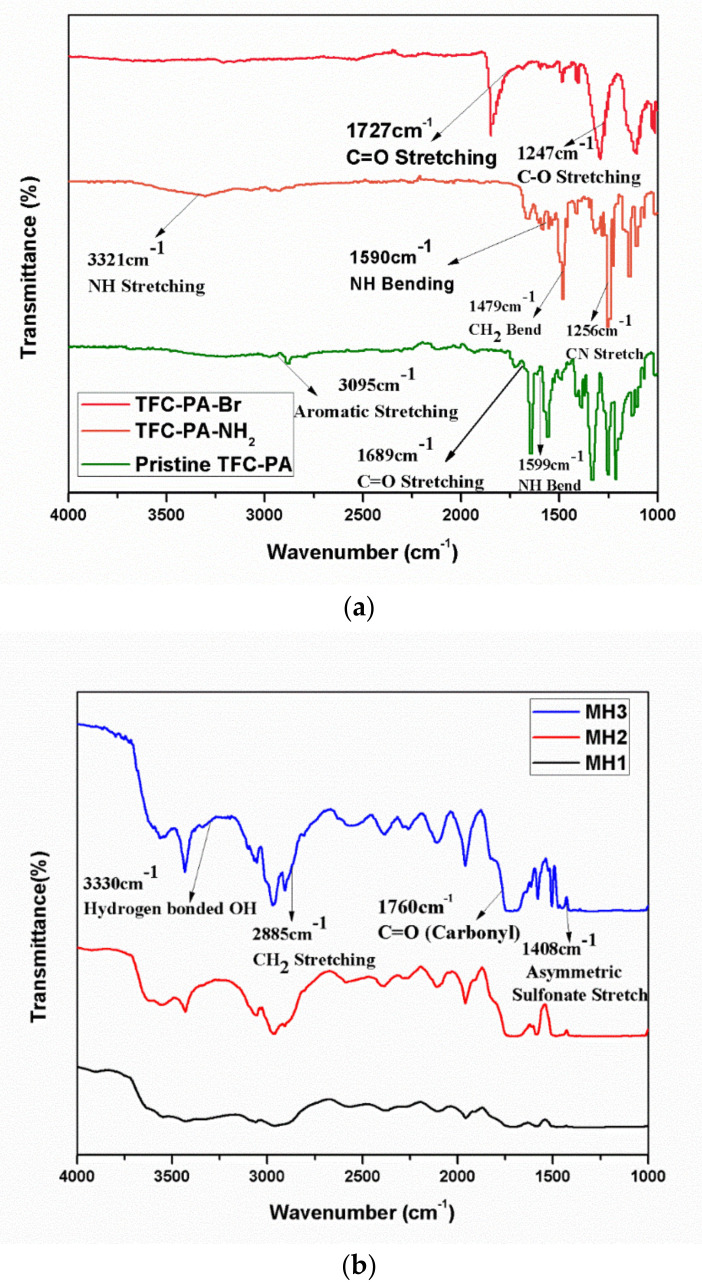
(**a**) FTIR of Pristine TFC PA, TFC PA-NH_2_, and TFC-PA-Br; (**b**) FTIR of modified membranes MH1, MH2, and MH3.

FTIR spectra of PA, PA-NH_2_, and PA-Br are shown in Figure 3a. Spectra of pristine polyamide are shown in Figure 3a, the peak at 3095 cm^−1^ shows the aromatic stretching, which is due to the aromatic group in polyamide, the peak at 1689 cm^−1^ is designated to C=O, and the peak at 1599 cm^−1^ is that for NH bending of the peptide bond present within the polyamide structure [28]. Figure 3a shows the spectra for PA-NH_2_ and PA-Br. In the PA-NH_2_ spectrum, the peak at 3321 cm^−1^ is assigned to N-H bond stretching due to the presence of an amine group; the broad peak results from weak N-H bond stretching, and the peak at 1590 cm^−1^ is due to the N-H bond bending [28]. A sharp peak shows that extensive bending has occurred in the bond. The peak at 1479 cm^−1^ indicates CH_2_ bend that represents the CH_2_ group present in the attached APTMS chain.

**Figure 4:**
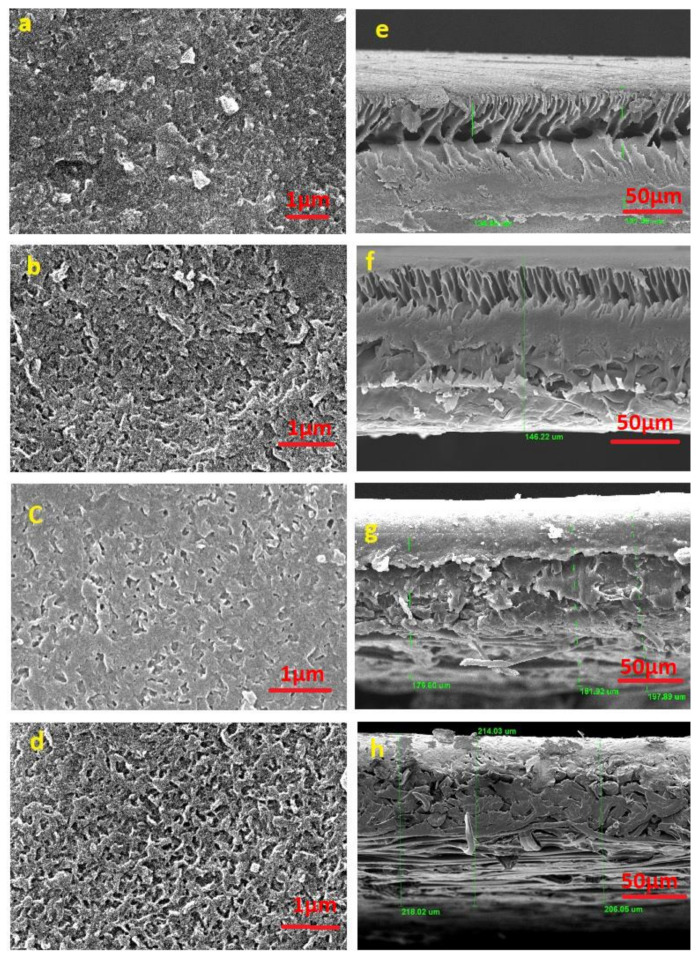
SEM images of surface morphology (**a**) Pristine, (**b**) MH1, (**c**) MH2, (**d**) MH3, SEM cross-section (**e**) Pristine, (**f**) MH1, (**g**) MH2, (**h**) MH3.

The spectra of PA-Br is shown in Figure 4a, and the peaks that are formed due to N-H stretching and bending have disappeared because of the conversion of PA-NH_2_ to PA-Br; moreover, the peak at 1727 cm^−1^ shows the presence of C=O in the attached ethyl α bromoisobutyrate [28].

The FTIR spectra of modified PA by grafting PSPMK show interesting chemical transformations in Figure 3b. The band at 1760 cm^−1^ is attributed to C=O stretching of the carbonyl group present in the PSPMK chains [17]. The small and broad band at 3330 cm^−1^ is due to the water molecules attached to the sulfonic acid group of PSPMK [30]. The band at 2885 cm^−1^ indicates CH2 stretching vibrations [31]. The bands at 1062 cm^−1^ and 1408 cm^−1^ indicate symmetric and asymmetric sulfonate stretch [32,33,34]. All these results strongly assist the successful grafting of hydrophilic PSPMK polymer brushes onto a PA membrane via ATRP by using the “Grafting from” approach.

### 3.3. Morphological Observation

Membrane morphology was analyzed by the images of scanning electron microscopy as shown in Figure 4. It was seen that the pristine membrane had a network surface similar to the rugged surface morphologies usually in polyamide membranes with “ridges and valleys” [35] (Figure 4a). A “smoother” surface is exhibited by the modified membranes (Figure 4c,e,g), showing a considerable difference to the pristine membrane [36,37]. The modified membrane surface is uniformly and completely covered by thick sulfonate polymer brushes synthesized by SI-ATRP. The thickness of sulfonate brushes increases due to swelling in humid environment [38]. Cross-sectional SEM images (Figure 4b,d,f,h) show the asymmetric finger-like growth of polymer brushes, implying that there were small micro-pores in the membrane and it has a dense structure [39]. The thickness of membrane ranges from 118 to 218 µm, which is consistent with the previous work [17].

### 3.4. Contact Angle Measurements

**Figure 5:**
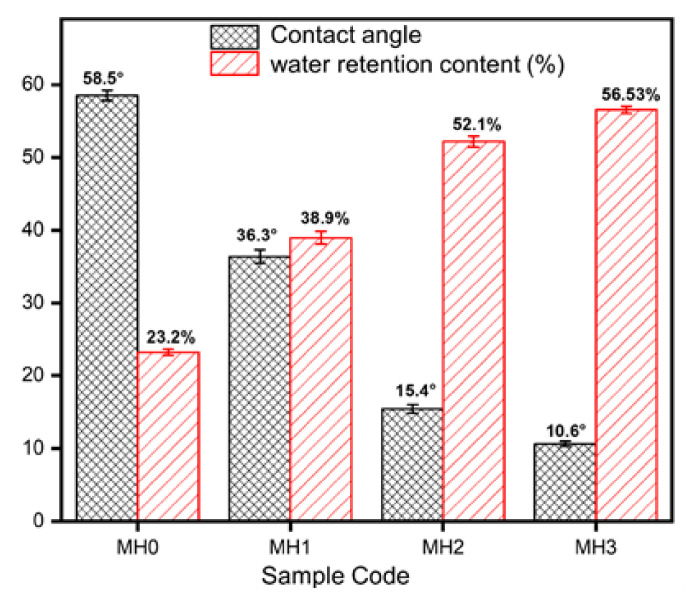
Graph showing the average contact angle values and percentage of water retention content of membrane samples.

The average contact angle and water content percentage of the modified membranes were measured to calculate the wettability of the membrane surface, and the results are summarized in Figure 5. The grafting of hydrophilic ionically charged PSPMK polymer brushes bearing negatively charged acrylate groups significantly increases the surface wettability of the membrane samples. For example, when the grafting of 4.06 mmol SPMK was conducted on MH1, it exhibits great variation in the contact angle of about 22.2° with a value of 36.3° as compared to MH0 membrane with a 58.5° value, while MH3 shows a contact angle value of 10.4° with a significant increase in wettability. The hydrophilic nature of grafted PSPMK polymer brushes evidently increases the hydrophilic character of the modified membrane [17]. In addition, the hydrophilic character of modified membranes is examined using water retention measurements. Water retention is interrelated with water contact angle [40]. Increased water retention content and a decreased contact angle contribute positively to improved membrane wettability [40,41]. In addition, the contact angle and interfacial property are linked with each other. A lower contact angle results in high interfacial tension for a hydrophilic membrane surface [42]. The MH0 membrane exhibits the highest contact angle of 58.5° and the lowest water retention content of 23.2%. The percentage of water retention content begins to increase from 38.9% to 52.1% by adding hydrophilic polymer brushes in MH1 and MH2 respectively, while MH3 expresses the enhanced water retention content of 56.53%. Therefore, the improvement in the grafting concentration of hydrophilic polymer brushes has a significant and beneficial influence on membrane hydrophilicity [43,44]. The minimum contact angle is shown by the PA-g-PSPMK as a result of hydrophilic ionically charged groups facilitating the passage of hydrophilic water molecules by hydrogen bonding and enhancing the wettability [45].

### 3.5. Surface Roughness

**Figure 6:**
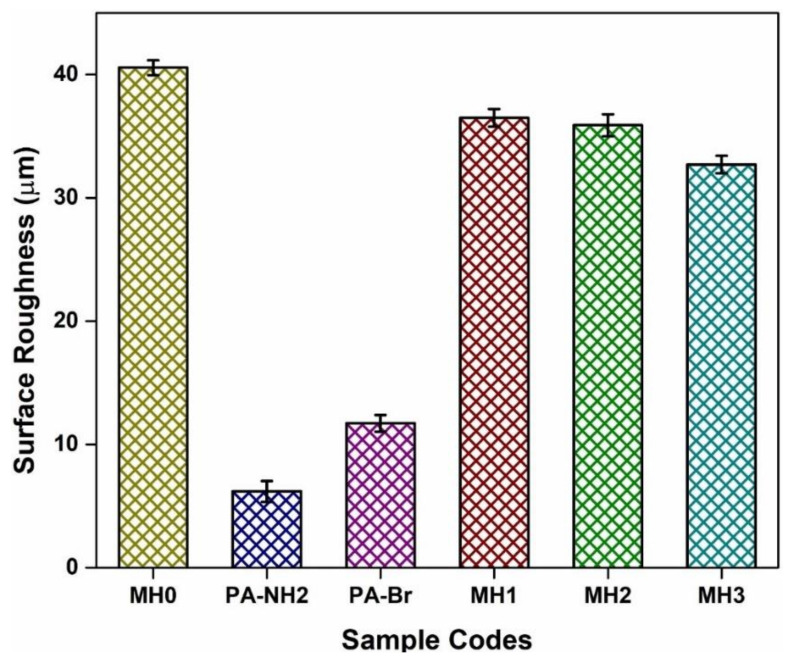
Graph showing the surface roughness values of all samples.

**Figure 7:**
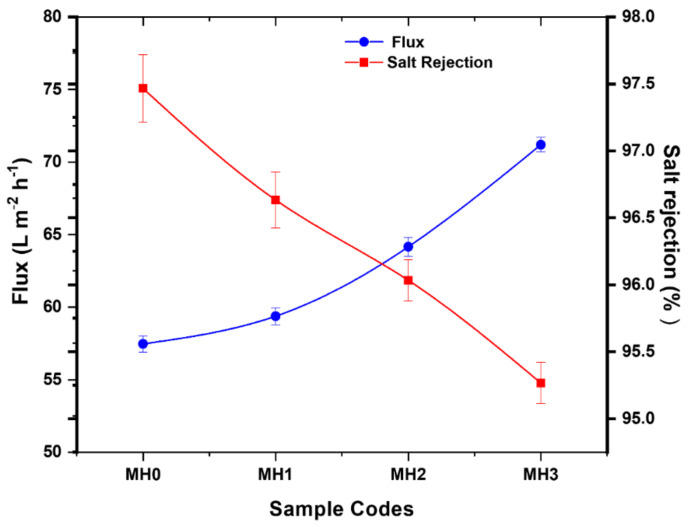
Permeation flux and salt rejection of pristine and modified membranes.

The roughness of the pristine and grafted membranes has been studied using optical profilometry. Roughness is the main predictor for calculating the degree of fouling of membranes [46]. Figure 6 shows the average roughness of membrane surfaces. The increased surface roughness results in more severe membrane fouling [20]. The grafting of PSPMK polymer brushes results in a smooth hydrophilic membrane surface [47]. A pristine PA membrane shows high roughness owing to the presence of ridge and valley-like morphology [48]. The PA-NH_2_ bar corresponds to the attachment of an amine group on the TFC-PA membrane. As a result of membrane functionalization, a uniform and even surface shows a decreased average surface roughness value that gradually rises after the attachment of bromo initiator represented by PA-Br bar [28]. With increasing polymer brush growth, the average surface roughness is reduced as a result of the polymer brush layer forming observed in the previous study [49]. In Figure 7, the pristine membrane has an average surface roughness of 40.559 µm, while PA-g-PSPMK has a value of 32.708 µm due to the growth of hydrophilic polymer brushes of PSPMK. This will favor the enhanced antifouling properties by making a more smooth surface than the simple TFC PA membrane [20]. A smoother surface inhibits the adhesion and settlement of foulants [50]. So, it can be concluded that due to the growth of uniform PSPMK brushes, the surface roughness has been decreased, which had a positive impact on the antibacterial and anti-biofilm characteristics of the membrane due to its smooth surface [51].

### 3.6. Evaluation of Membrane Performance

Custom-made filtrate assembly was used to determine the performance of the membrane. The water flux and salt rejection efficiency was calculated at 1.0 MPa with feed water containing 2000 ppm NaCl, and the results are shown in Figure 7. The pristine membrane (MH0) showed the minimum value of water flux, which was 57.4 L m^−2^ h^−1^ due to the hydrophobic nature of the PA membrane in it [28]. The maximum flux was 7l.2 L m^−2^ h^−1^ for MH3 at 12.185 mmol PSPMK concentration. An increasing PSPMK concentration has been observed to increase permeability flow. The hydrophilicity of the TFC-PA-grafted PSPMK has been considerably increased due to the presence of PSPMK [28]. It has already been described that the increase in surface hydrophilicity has a positive impact on permeability flux [52]. The current finding is compatible with the research done earlier suggesting that water is moving through the membrane by establishing channels of water in the presence of hydrophilic ionic groups by avoiding salt movement [16]. The hydrophilicity of a TFC PA-grafted PSPMK was considerably improved by the inclusion of the hydrophilic PSPMK, according to the contact angle and surface roughness. As reported earlier, inter-chain hydrogen bonding enhances the hydrophilic character of PSPMK polymer brushes, due to which more water molecules are attracted toward the membrane [45].

Sodium chloride (NaCl) rejection is an adequate consideration to determine the performance of high-pressure reverse osmosis membrane. The pristine membrane showed 97% salt rejection with a water flux of 57.4 L m^−2^ h^−1^. The MH3 membrane, on the other hand, showed a flux of 71.2 L m^−2^ h^−1^ with an NaCl rejection of 95.2% ± 1.8 for MH3 (Figure 7). MH3 shows slight decrease in salt rejection but is in close proximity to the pristine RO membrane. The salt rejection decreases slightly because of the elevation in the flux rate, as the membrane requires optimization between salt rejection and permeability flux while operational [53].

Table 2 shows numerous research reports suggesting that the grafting of polymer brushes affects the flux rate and salt rejection. Rana et al. copolymerizes SPMK with methylene-bis-acrylamide (MBA) in the presence of cerium (IV)/polyvinyl alcohol (PVA) exhibiting a flux rate of 62.9 L m^−2^ h^−1^ and 94% salt rejection [33]. In another study, Xu et al. reported the modification of the TFC PA membrane by depositing chitosan (CS) on the surface of the membrane. It showed a water flux of 57.7 L m^−2^ h^−1^ with a salt rejection of 95% [54]. Zhang et al. grafted poly sulfobetaine methacrylate on a TFC-PA-RO membrane by SI-ATRP showing a flux rate of 37 L m^−2^ h^−1^ with an ionic rejection of 92% [55]. In a recent report, TFC RO membrane was modified with poly (amide urethane imide) (PAUI) loaded with silver (Ag), indicating a flux rate of 40 L m^−2^ h^−1^ and salt rejection between 90 and 95% [56]. In the current study, the TFC-PA-RO membrane was modified by grafting PSPMK polymer brushes significantly exhibiting enhanced water flux and salt rejection along with biologically stable fouling resistant properties.

### 3.7. Antibacterial Activity

**Figure 8:**
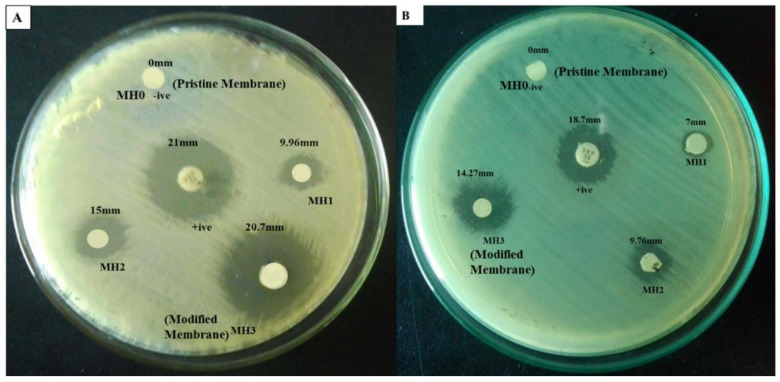
Antibacterial activity of PA g PSPMK Membranes against (**A**) *E. coli* and (**B**) *S.aureus.*

**Figure 9:**
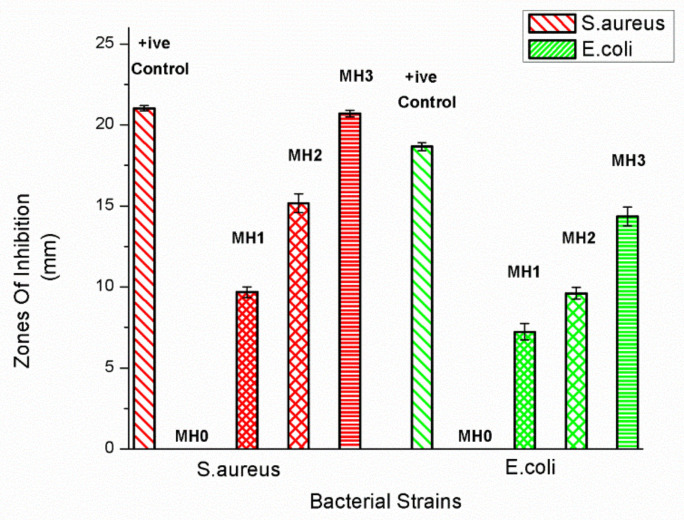
Graph showing inhibition zones against *E. coli* and *S. aureus* by pristine membrane and PSPMK-modified membranes.

The antibacterial effects of the TFC-RO membrane grafted with PSPMK were evaluated by the disc diffusion method and the average inhibition zones are presented in Figure 8 and Figure 9. Prominent zones of inhibition were shown by all test strains, indicating the positive results in case of three different concentrations of SPMK. After measuring the antibacterial inhibition zones, it was found that the pristine PA membrane has no significant inhibition zones and bacterial colonies has been scattered all over the membrane surface. Bacterial adhesion and settlement was mainly related to the hydrophobicity, surface roughness, and electrostatic charges that are conferred by different macromolecules present on a membrane surface [20,57,58,59]. The rupture of a bacterial cell wall by an increasing grafting concentration of anionic, sulfonated, hydrophilic polymer, and electrostatic repulsive forces plays a vital role in forming significant inhibition zones as the concentration of PSPMK increases [60]. In case of *E. coli*, the reason behind the formation of inhibition zones is the negatively charged outer surface of a Gram-negative bacterium (*E. coli*) repelled by the negatively charged surface of modified membrane due to repulsive electrostatic interactions [61]. While for *S.aureus,* discs of the modified membrane labeled as MH1, MH2, and MH3 show inhibition zones as the concentration of sulfonated, hydrophilic, water swell ability, and anionic polymer increases [60]. The sulfonated group promotes polymer hydration and pH reduction, which applies stress on the outer membrane, destroying it, accompanying microbial death, enzyme disruption, and protein denaturation [62]. The results suggested that the PA g PSPMK can be recommended as a promising material for protecting the TFC RO membrane against bacterial attack.

### 3.8. Resistance to Biofilm Formation

**Figure 10:**
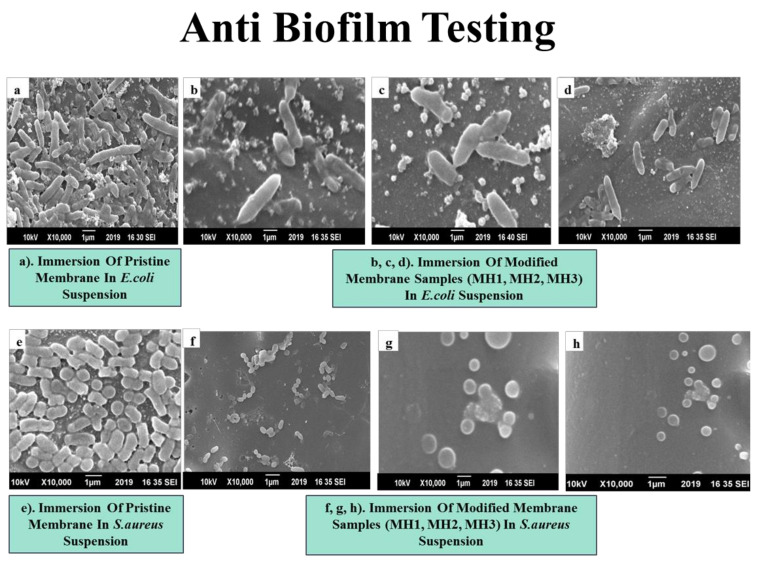
SEM images of the membrane samples after immersion in bacterial suspension (**a**) Pristine membrane in *E. coli* (**b**)–(**d**). Modified membrane in *E. coli* suspension (**e**). Pristine membrane in *S. aureus* suspension (**f**)–(**h**). Modified membrane in *S. aureus* suspension.

A Gram-negative bacterium *E. coli* and a Gram-positive bacterium *S.aureus,* because of their rich existence in water, have been used to evaluate the resistance to biofilm formation on a commercial TFC-PA RO membrane. Figure 10 shows the SEM images of membrane samples immersed in a bacterial suspension for 72 h. For the pristine membrane (Figure 10a), the rod-shaped *E. coli* bacteria were evenly distributed all over the surface. The reason behind the accumulation of bacteria on a pristine PA surface was hydrophobic interactions, as reported earlier that the accumulation of bacteria prevails by two factors such as hydrophilic/hydrophobic interactions and electrostatic forces [63,64]. In Figure 10b–d, only a few *E. coli* bacteria were attached to the poly anionic PSPMK-grafted PA membrane due to the presence of repulsive electrostatic interactions. It has been noted that the development of biofilm over a negatively charged surface is not permanent and can be quickly separated from a negatively charged surface, with a decrease in bacterial motility compared to polycationic surfaces [65]. Therefore, the adhesion of bacteria and biofilm formation on a negatively charged surface was effectively reduced due to electrostatic repulsion [58].

In Figure 10e, the surface of unmodified PA membrane was densely covered by *Staph.aureus* bacteria due to hydrophobic interactions. Whereas, in Figure 10f–h, for PA g PSPMK hydrophilic modified membrane, only a few bacteria were present, lacking the hydrophobic interactions between the membrane surface and microbial surface [65]. The hydration of hydrophilic brushes has been documented to provide an unfavorable atmosphere for the fixation of bacterial cells [51]. The grafting of hydrophilic, sulfonated anionic polymer brushes ruptures the outer membrane of bacteria, resulting in the damage of bacterial enzymes [66,67]. Therefore, it can be concluded that upon the hydration of grafted sulfonate, the PSPMK brush applies stress on the outer membrane surface, destroying the membrane, resulting in enzyme damage and protein denaturation ultimately causing the death of microbes [68].

## 4. Conclusions

Hydrophilic, anionic polysulfopropyl methacrylate polymer brushes were prepared and grafted with different concentrations into the polyamide to fabricate a highly hydrophilic, antifouling reverse osmosis polymeric membrane. The modified membrane was analyzed for its utilization in water disinfection. The PA g PSPMK was prepared and characterized by FTIR, SEM, and optical profilometry. Immobilization of the EBIB initiator onto the functionalized polyamide membrane was evident by the interpretation of FTIR spectra. The TFC-PA-RO membranes grafted by varying the PSPMK concentrations of 4.06 mmol, 8.12 mmol, and 12.18 mmol were modified by using the “grafting from” approach via ATRP. The membrane characterization shows a remarkable effect on the membrane surface properties such as hydrophilicity, ruggedness, and membrane flux as the SPMK concentrations increase. The fouling examination of membrane samples revealed a decline of the colony of bacteria. It was observed that the membrane with a maximum concentration of 12.18 mmol demonstrates the considerable lowering in the bacterial colony among all the PSPMK-modified membranes. The results disclose that the modification in polymer membranes can be an effective approach to overcome the severe issue of biofouling leading to water treatment through membranes.

## Figures and Tables

**Table 1:** Feed ratios of reagents used for synthesis of 3-sulfopropyl methacrylate potassium (SPMK) using atom transfer radical polymerization (ATRP) in dimethylformamide (DMF): H_2_O solvent at 40 °C.

Material for ATRP	Membrane Sample Codes		
	MH1	MH2	MH3
**SPMK (mmol)**	4.06	8.12	12.185
**DMF (mL)**	1.25	2.50	3.75
**Water (mL)**	0.75	1.5	2.25
**Bipy (mmol)**	2.0	3.248	4.874
**CuBr I (mmol)**	0.4	0.649	0.974
**CuBr II (mmol)**	0.4	0.649	0.974
**EBIB (mmol)**	0.1	0.162	0.243

**Table 2:** Comparison between current work and previous studies.

Monomers Grafted on TFC-PA Membrane	Feed Pressure (MPa)	Flux L m^−2^ h^−1^	Salt Rejection (%)	References
PSPMA	1.0	71.2	95.2 ± 1.8	Present
PA g poly(SPMA co MBA) g PVA	1.72	62.9	94	[36]
PA/CS	0.8	57.7	95	[54]
pSBMA	4.5	37	92	[55]
Ag loaded PAUI	1.55	40	90–95	[56]
